# Association of polymorphisms in *FBN1*, *MYH11*, and TGF-β signaling-related genes with susceptibility of sporadic thoracic aortic aneurysm and dissection in the Zhejiang Han population

**DOI:** 10.1515/med-2024-1025

**Published:** 2024-09-13

**Authors:** Shasha Yu, Lujie Huang, Jianfei Ren, Xiaoying Zhang

**Affiliations:** Ningbo Medical Center Lihuili Hospital, Zhejiang, China; Ningbo Medical Center Lihuili Hospital, 57 Xingning Road, Zhejiang, China

**Keywords:** sporadic thoracic aortic aneurysm and dissection, polymorphism, FBN1, MYH11, TGF-β, the Chinese population

## Abstract

**Background:**

Sporadic thoracic aortic aneurysm and dissection (sTAAD) is a complicated vascular disease with a high mortality rate. And its genetic basis has not been fully explored.

**Method:**

Here, 122 sTAAD patients and 98 healthy individuals were recruited, and 10 single nucleotide polymorphisms were selected and analyzed (FBN1 rs10519177, rs1036477, rs2118181, MYH11 rs115364997, rs117593370, TGFβ1 rs1800469, TGFβ2 rs900, TGFβR2 rs764522, rs1036095, and rs6785385). Moreover, multiple logistic regression analysis was used to evaluate gene–environment interactions.

**Results:**

We identified that TGFβR2 rs1036095 dominant model CC + CG genotype (GT) (*P* = 0.004) may be a factor of increased risk of sTAAD, especially for women. FBN1 rs1036477 recessive model AA GT (*P* = 0.009) and FBN1 rs2118181 dominant model CC + CT GT (*P* = 0.009) were correlated to an increased death rate in sTAAD men patients. Gene–environment interactions indicated TGFβR2 rs1036095 dominant model (CC + CG)/GG to be a higher-risk factor for sTAAD (odds ratio = 3.255; 95% confidence interval: 1.324–8.000, *P* = 0.01).

**Conclusions:**

TGFβR2 rs1036095, FBN1 rs1036477, and FBN1 rs2118181 were identified as factors of increased risk of sTAAD. Gene–environment interactions were associated with the risk of sTAAD.

## 1 Introduction

Thoracic aortic aneurysm and dissection (TAAD) is a debilitating disorder with a high mortality rate due to its rapid advancement [1]. The primary pathological basis of TAAD is the depletion of smooth muscle cells (SMCs), destruction of the extracellular matrix (ECM), and inflammation, which are caused by gene mutations [2,3,4]. To date, the relationship between the pathogenesis of familial TAAD and associated gene mutations has been identified in various genetic diseases, including Marfan syndrome (MFS) and Loeys–Dietz syndrome (LDS) [3,5,6]. Notably, TAAD-related gene mutations exhibit extensive heterogeneity. It is reported that over 40 genes are reportedly associated with TAAD. Currently, the genes identified to be involved in the development of aortic aneurysms are members of various protein systems, including ECM regulation (*FBN1*/*2*), vascular smooth muscle cell (VSMC) contractile apparatus (*MYH11* and *ACTA2*), and transforming growth factor β (TGF-β) signaling (*TGF*β*R1*/*2*). These protein systems are important and fundamental factors in the progression of aortic disorders [7,9].

Fibrillin-1, encoded by *FBN1*, is a necessary structural component of ECM microfibrils. It is noteworthy that *FBN1* is associated with MFS, in which TAA is a clinical symptom [10]. A genetic study designated a genome-wide association study has lately discovered genetic polymorphisms at 15q21.1, which is situated within the coding sequence of *FBN1*. These single-nucleotide polymorphisms (SNPs), specifically rs2118181, and rs10519177, have been demonstrated to be associated with TAAD in previous studies [7,11]. Similarly, the *FBN1* SNPs rs2118181 and rs1036477 were identified as risk factors for the development of ascending aortic dissection (AD) in the Lithuanian population in a study by Lesauskaite.

The protein denoted as Myosin Heavy Chain Protein 11 (*MYH11*) is a contractile protein that is specifically expressed in SMCs. The aortic vascular middle membrane is composed of VSMCs and ECM. Dysfunction in SMC contraction is a critical factor that contributes to the development of AD [12,13]. Mutations in *MYH11* can lead to familial TAAD, among which most patients have patent ductus arteriosus [14,15].

Furthermore, there is a strong correlation between the TGF-β pathway and the development of AD [16]. Increased levels of TGF-β expression caused by variations in TGF-β pathway-related genes contribute to the transformation of contractile VSMCs into synthetic VSMCs in human aortic vessels, stimulate the increased synthesis of collagen by arterial SMCs, and disrupt the balance between the aortic wall structure and ECM [17,18]. Heterozygous mutations in the TGF-β type I and II receptors TGFβR1/2 initiate steps in the pathogenesis of AD or aneurysm [19]. In particular, mutations in the TGFβR1/2 gene have been identified in patients with TAAD and LDS, which are characterized by reduced smooth muscle contractility, whereas high expression of Smad2/3/4 has been observed [20]. Overall, mutations in TGF-β pathway-related genes result in aberrant TGF-β signal conduction, leading to phenotypic alterations in VSMCs, and ultimately contributing to the development of TAAD [21].

Notably, various groups have reported that the pathogenesis of AD is influenced by factors such as FBN1, MYH11, and the TGF-β pathway. However, previous studies related to the role of FBN1, MYH11, and the TGF-β signaling pathway in AD have mainly focused on familial TAAD and aortic syndromes, such as MFS and LDS. Furthermore, the occurrence of AD is primarily limited to sporadic cases. While the majority of TAAD cases are sporadic, the genetic basis of Sporadic thoracic aortic aneurysm and dissection (sTAAD), especially in the Zhejiang Han population, remains largely uninvestigated.

Consequently, herein, the correlation between polymorphisms in MYH11, FBN1, and TGF-β pathway-related genes and sTAAD susceptibility in the Zhejiang Han population was explored.

## 2 Materials and methods

### 2.1 Participants

A case–control study concerning the Han population from the Zhejiang Province was performed, and 122 TAAD patients were recruited from the Ningbo Medical Center Lihuili Hospital between January 2019 and December 2020. The condition of each patient was confirmed by aortic CTA or thoracic endovascular aortic repair treatment. Simultaneously, a control group of 98 healthy individuals from the same hospital’s health clinic was recruited. Patients with connective tissue disease, cancer, or other malignant diseases, or familial TAAD were excluded from our study.

### 2.2 Genotyping

Information about the 10 tag SNPs was obtained from the dbSNP NCBI and UCSC Genome Browser website, which is located at http://genome.ucsc.edu/. The study employed standard linkage disequilibrium patterns with *r* ^2^ > 0.8 and a minor allele frequency of >0.05.

Blood samples were collected from all participants by venipuncture. The Tiangen DNA Extraction Kit was used for extracting genomic DNA from isolated peripheral vein blood leukocytes. SNPs were amplified and genotyped using polymerase chain reaction and sequencing, respectively. Genotype (GT) analysis was performed on randomly selected (5%) samples using a blinded method, with 100% consistency.

### 2.3 Statistical analysis

The SPSS software (version 26.0; SPSS Inc., USA) was applied for all analyses. Measurement data are reported as mean ± standard deviation. Comparison between the two groups (case and control participants) was performed by the independent-sample *t*-test. Meanwhile, using the *x* ^2^ test, the frequency distribution of GTs and alleles was evaluated from the Hardy–Weinberg equilibrium (HWE). The traditional TAAD risk factors were analyzed by the multivariate unconditional logistic regression analysis, expressing the risks by odds ratio (OR) and 95% confidence interval (CI). The value of *P* < 0.05 was deemed a significant difference.


**Ethical approval:** This study was approved by the ethics committee of the Ningbo Medical Center Lihuili Hospital and conducted according to the principles set by the Declaration of Helsinki.
**Informed consent:** All participants provided a signed informed consent form.

## 3 Results

### 3.1 Characteristics of study participants

A total of 220 patients were enlisted in this study. This included 122 patients with sTAAD (96 men and 26 women; the average age of 60.35 ± 13.40 years) and 98 healthy participants as control (81 men and 17 women; age on average of 51.76 ± 14.64 years), revealing that sTAAD patients exhibited a statistically significant increased risk of hypertension and smoking when compared to the control (*P* < 0.05). Additionally, it was detected that individuals in the sTAAD group exhibited higher systolic and diastolic blood pressure and more elevated creatinine levels, whereas lower levels of total cholesterol (TC), triglycerides (TG), high-density lipoprotein-cholesterol (HDL-C), and low-density lipoprotein-cholesterol (LDL-C) when compared to the control (*P* < 0.05). Nevertheless, the terms blood glucose, alcohol consumption, age, and sex had no significant difference between the two groups (Table 1).

**Table 1:** General characteristics between case and control subjects

Char	Case (122)	Control (98)	*P*
Age	60.35 ± 13.40	51.76 ± 14.64	0.231
Male (*n*, %)	96 (78.6)	81 (82.7)	0.068
Hypertension (*n*, %)	88 (72.1)	26 (26.5)	<0.001*
Diabetes (*n*, %)	10 (8.1)	6 (6.1)	0.556
Smoking (*n*, %)	46 (37.7)	22 (22.4)	0.015*
Drinking (*n*, %)	30 (24.6)	14 (14.2)	0.058
SBP (mmHg)	135.00 (120.75-150.25)	126.00 (118.00–134.50)	<0.001*
DBP (mmHg)	80.00 (69.75–88.25)	72.00 (68.00–78.00)	<0.001*
Creatinine (μmol/L)	73.00 (57.75–90.25)	67.00 (58.00–77.00)	<0.001*
Triglyceride (mmol/L)	1.11 (0.79–1.53)	1.46 (0.96–2.17)	<0.001*
Total cholesterol (mmol/L)	4.22 ± 1.01	5.25 ± 0.80	<0.001*
HDL-C (mmol/L)	1.08 (0.90–1.22)	1.22 (1.03–1.44)	<0.001*
LDL-C (mmol/L)	2.35 (1.93–2.87)	3.26 (2.87–3.75)	<0.001*
Aorta diameter (mm)	36.93 ± 4.63		

*Significant *P*-values (a *P*-value of less than 0.05).

SBP, systolic blood pressure; DBP, diastolic blood pressure; HDL-C, high-density lipoprotein cholesterol; LDL-C, low-density lipoprotein cholesterol.

### 3.2 GFs and allele frequencies (AFs)

Table 2 shows SNP GFs and AFs distributions in both sTAAD and control groups, revealing ten SNP GFs in the case and control participants according to HWE (*P* > 0.05). Differences were detected not only in the GFs of TGFβR2 rs1036095 (*P* = 0.006), FBN1 rs1036477 (*P* = 0.029), and FBN1 rs2118181 (*P* = 0.029) but also in the AFs of TGFβR2 rs1036095 (*P* = 0.001), FBN1 rs1036477 (*P* = 0.007), and FBN1 rs2118181 (*P* = 0.007) among the two groups. However, no significant differences were detected between GTs and alleles of FBN1 rs10519177, MYH11 rs115364997, MYH11 rs117593370, TGFB1 rs1800469, TGFβ2 rs900, TGFβR2 rs764522, and TGFβR2 rs6785385 among the two groups (*P* > 0.05).

**Table 2:** Description for GT and AFs in the case and control groups

SNP	GT/allele	Case, *n* (%)	Control, *n* (%)	*p*
TGF-β1 rs1800469	AA	28	20	0.763
	AG	62	54	
	GG	31	22	
	A	118	94	0.967
	G	124	98	
TGF-β2 rs900	AA	8	5	0.55
	AT	45	43	
	TT	68	49	
	A	61	53	0.618
	T	181	141	
TGFBR2 rs764522	CC	93	79	0.249
	CG	26	14	
	GG	2	4	
	C	212	172	0.735
	G	30	22	
TGFBR2 rs1036095	CC	8	1	0.006*
	CG	39	19	
	GG	73	76	
	C	55	21	0.001*
	G	185	171	
TGFBR2 rs6785385	AA	2	4	0.249
	AG	29	29	
	GG	91	63	
	A	33	37	0.105
	G	211	155	
FBN-1 rs10519177	AA	53	35	0.216
	AG	52	41	
	GG	15	20	
	A	158	111	0.087
	G	82	81	
FBN-1 rs1036477	AA	84	51	0.029*
	AG	31	38	
	GG	5	8	
	A	199	140	0.007*
	G	41	54	
FBN-1 rs2118181	CC	5	8	0.029*
	CT	31	38	
	TT	84	51	
	C	41	54	0.007*
	T	199	140	
MYH11 rs117593370	CC	119	93	0.702
	CT	3	4	
	TT	0	0	
	C	241	190	0.705
	T	3	4	
MYH11 rs115364997	AA	96	79	0.494
	AG	24	13	
	GG	2	1	
	A	216	171	0.243
	G	28	15	

*Significant *P*-values (a *P*-value of less than 0.05).

### 3.3 Analysis of genetic models and the risk of sTAAD

We further assessed the association between genetic models and the risk of sTAAD (Table 3). Notably, it was observed that the TGFβR2 rs1036095 dominant model CC + CG GT (OR = 2.447; 95% CI: 1.324–4.521, *P* = 0.004), FBN1 rs1036477 recessive model AA GT (OR = 2.105; 95% CI: 1.205–3.677, *P* = 0.009), and FBN1 rs2118181 dominant model CC + CT GT (OR = 0.475; 95% CI: 0.272–0.830, *P* = 0.009) were sTAAD risk factors. In addition, there was no statistically significant correlation of other SNP GTs to the risk of sTAAD (*P* > 0.05).

**Table 3:** Analysis of the association between genetic models and sporadic TAAD risk

SNP	Genetic model	GT	OR	95% CI	*p*
TGF-β1 rs1800469	Dominant	(AA + AG)/GG	0.863	0.461–1.616	0.645
	Recessive	AA/(AG + GG)	1.144	0.598–2.189	0.684
	Additive	AA	1		
		AG	0.820	0.415–1.619	0.568
		GG	1.006	0.456–2.223	0.987
TGF-β2 rs900	Dominant	(AA + AT)/TT	0.796	0.465–1.360	0.403
	Recessive	AA/(AT + TT)	1.303	0.412–4.117	0.652
	Additive	AA	1		
		AT	0.654	0.198–2.156	0.485
		TT	0.867	0.268–2.812	0.813
TGFBR2 rs764522	Dominant	(CC + CG)/GG	2.559	0.459–14.276	0.284
	Recessive	CC/(CG + GG)	0.757	0.390–1.470	0.411
	Additive	CC	1		
		CG	1.578	0.771–3.227	0.212
		GG	0.425	0.076–2.381	0.33
TGFBR2 rs1036095	Dominant	(CC + CG)/GG	2.447	1.324–4.521	0.004*
	Recessive	CC/(CG + GG)	0.147	0.018–1.200	0.073
	Additive	CC	1		
		CG	0.257	0.030–2.203	0.215
		GG	0.12	0.015–0.984	0.048*
TGFBR2 rs6785385	Dominant	(AA + AG)/GG	0.65	0.362–1.169	0.15
	Recessive	AA/(AG + GG)	0.383	0.069–2.139	0.274
	Additive	AA	1		
		AG	2	0.339–11.785	0.444
		GG	2.889	0.513–16.255	0.229
FBN-1 rs10519177	Dominant	(AA + AG)/GG	1.842	0.886–3.829	0.102
	Recessive	AA/(AG + GG)	1.379	0.795–2.390	0.253
	Additive	AA	1		
		AG	0.838	0.464–1.513	0.557
		GG	0.495	0.224–1.096	0.083
FBN-1 rs1036477	Dominant	(AA + AG)/GG	2.067	0.654–6.537	0.216
	Recessive	AA/(AG + GG)	2.105	1.205–3.677	0.009*
	Additive	AA	1		
		AG	0.495	0.275–0.892	0.019*
		GG	0.379	0.118–1.223	0.105
FBN-1 rs2118181	Dominant	(CC + CT)/TT	0.475	0.272–0.830	0.009*
	Recessive	CC/(CT + TT)	0.484	0.153–1.529	0.216
	Additive	CC	1		
		CT	1.305	0.388–4.394	0.667
		TT	2.635	0.818–8.493	0.105
MYH11 rs117593370	Dominant	(CC + CT)/TT	—		
	Recessive	CC/(CT + TT)	1.706	0.373–7.811	0.491
	Additive	CC	1		
		CT	0.586	0.128–2.683	0.491
		TT	—		
MYH11 rs115364997	Dominant	(AA + AG)/GG	0.652	0.058–7.303	0.729
	Recessive	AA/(AG + GG)	0.654	0.320–1.337	0.245
	Additive	AA	1		
		AG	1.519	0.727–3.177	0.267
		GG	1.646	0.147–18.488	0.686

*Significant *P*-values (a *P*-value of less than 0.05).

### 3.4 GFs and AFs after gender stratification

Additionally, GFs and the risk of sTAAD were examined after performing stratification according to sex (Tables 4 and 5), identifying both the GFs of TGFβR2 rs6785385 (*P* = 0.039), FBN1 rs10519177 (*P* = 0.015), FBN1 rs1036477 (*P* = 0.001), and FBN1 rs2118181 (*P* = 0.001), besides the AFs of TGFBR2 rs6785385 (*P* = 0.012), FBN1 rs10519177 (*P* = 0.005), FBN1 rs1036477 (*P* = 0.001), and FBN1 rs2118181 (*P* = 0.001) in men differed between the two groups. Consistently, the GFs of TGFβR2 rs1036095 (*P* = 0.012) and AFs of TGFβR2 rs1036095 (*P* = 0.003) differed significantly in women between the sTAAD and control groups.

**Table 4:** Description for GT and AFs in men samples

SNP	GT/allele	Case, *n* (%)	Control, *n* (%)	*P*
TGF-β1 rs1800469	AA	20	9	0.625
	AG	50	35	
	GG	25	16	
	A	90	53	0.582
	G	100	67	
TGF-β2 rs900	AA	6	3	0.377
	AT	33	28	
	TT	56	30	
	A	45	34	0.407
	T	145	88	
TGFBR2 rs764522	CC	76	50	0.232
	CG	18	8	
	GG	1	3	
	C	170	108	0.793
	G	20	14	
TGFBR2 rs1036095	CC	5	1	0.172
	CG	30	13	
	GG	59	46	
	C	40	15	0.05
	G	148	105	
TGFBR2 rs6785385	AA	1	4	0.039*
	AG	22	20	
	GG	73	36	
	A	24	28	0.012*
	G	168	92	
FBN-1 rs10519177	AA	47	16	0.015*
	AG	36	32	
	GG	11	12	
	A	130	64	0.005*
	G	58	56	
FBN-1 rs1036477	AA	69	27	0.001*
	AG	22	30	
	GG	3	4	
	A	160	84	0.001*
	G	28	38	
FBN-1 rs2118181	CC	3	4	0.001*
	CT	22	30	
	TT	69	27	
	C	28	38	0.001*
	T	160	84	
MYH11 rs117593370	CC	93	59	0.957
	CT	3	2	
	TT	0	0	
	C	189	120	0.958
	T	3	2	
MYH11 rs115364997	AA	75	49	0.503
	AG	19	7	
	GG	2	1	
	A	169	105	0.259
	G	23	9	

*Significant *P*-values (a *P*-value of less than 0.05).

**Table 5:** Description for GT and AFs in women samples

SNP	GT/allele	Case, *n* (%)	Control, *n* (%)	*P*
TGF-β1 rs1800469	AA	8	11	0.797
	AG	12	19	
	GG	6	6	
	A	28	41	0.732
	G	24	31	
TGF-β2 rs900	AA	2	2	0.923
	AT	12	15	
	TT	12	19	
	A	16	19	0.593
	T	36	53	
TGFBR2 rs764522	CC	17	29	0.401
	CG	8	6	
	GG	1	1	
	C	42	64	0.205
	G	10	8	
TGFBR2 rs1036095	CC	3	0	0.012*
	CG	9	6	
	GG	14	30	
	C	15	6	0.003*
	G	37	66	
TGFBR2 rs6785385	AA	1	0	0.641
	AG	7	9	
	GG	18	27	
	A	9	9	0.453
	G	43	63	
FBN-1 rs10519177	AA	6	19	0.013*
	AG	16	9	
	GG	4	8	
	A	28	47	0.199
	G	24	25	
FBN-1 rs1036477	AA	15	24	0.62
	AG	9	8	
	GG	2	4	
	A	39	56	0.718
	G	13	16	
FBN-1 rs2118181	CC	2	4	0.62
	CT	9	8	
	TT	15	24	
	C	13	16	0.718
	T	39	56	
MYH11 rs117593370	CC	26	34	0.505
	CT	0	2	
	TT	0	0	
	C	52	70	0.509
	T	0	2	
MYH11 rs115364997	AA	21	30	0.794
	AG	5	6	
	GG	0	0	
	A	47	66	0.804
	G	5	6	

*Significant *P*-values (a *P*-value of less than 0.05).

### 3.5 Multiple logistic regression analysis for sTAAD

Multiple logistic regression analyses were utilized for evaluating the correlation between sTAAD development and its risk factors (hypertension, diabetes, smoking, TC, HDL-C, LDL-C, and the identified dominant model of three SNPs), revealing that hypertension, as well as TG, HDL-C, and LDL-C levels, were sTAAD risk factors(Table 6). After excluding confounding factors, the TGFBR2 rs1036095 dominant model (CC + CG)/GG was validated to be an important sTAAD risk factor (OR = 3.255; 95% CI: 1.324–8.000, *P* = 0.01).

**Table 6:** Multiple logistic regression analysis for sporadic TAAD

SNP	Risk factor	OR	95% CI	*P*
TGFBR2 rs1036095	Dominant (CC + CG)/GG	3.255	1.324–8.000	0.01*
	Hypertension (*n*, %)	6.43	2.751–15.031	<0.001*
	Diabetes (*n*, %)	0.725	0.128–4.105	0.716
	Smoking (*n*, %)	1.582	0.594–4.216	0.359
	Drinking (*n*, %)	0.684	0.217–2.155	0.517
	Creatinine (μmol/L)	1.012	0.989–1.034	0.309
	Triglyceride (mmol/L)	0.391	0.213–0.718	0.002*
	Total cholesterol (mmol/L)	1.938	0.727–5.169	0.186
	HDL-C (mmol/L)	0.086	0.012–0.604	0.014*
	LDL-C (mmol/L)	0.067	0.020–0.232	<0.001*
FBN-1 rs1036477	Recessive AA/(AG + GG)	2.036	0.930–4.459	0.075
	Hypertension (*n*, %)	7.019	3.062–16.091	<0.001*
	Diabetes (*n*, %)	0.765	0.151–3.887	0.747
	Smoking (*n*, %)	1.434	0.543–3.791	0.467
	Drinking (*n*, %)	0.622	0.202–1.920	0.409
	Creatinine (μmol/L)	1.014	0.991–1.037	0.235
	Triglyceride (mmol/L)	0.416	0.228–0.759	0.004*
	Total cholesterol (mmol/L)	1.75	0.665–4.601	0.257
	HDL-C (mmol/L)	0.083	0.012–0.587	0.013*
	LDL-C (mmol/L)	0.088	0.027–0.291	<0.001*
FBN-1 rs2118181	Dominant (CC + CT)/TT	0.491	0.224–1.075	0.075
	Hypertension (*n*, %)	7.019	3.062–16.091	<0.001*
	Diabetes (*n*, %)	0.765	0.151–3.887	0.747
	Smoking (*n*, %)	1.434	0.543–3.791	0.467
	Drinking (*n*, %)	0.622	0.202–1.920	0.409
	Creatinine (μmol/L)	1.014	0.991–1.037	0.235
	Triglyceride (mmol/L)	0.416	0.228–0.759	0.004*
	Total cholesterol (mmol/L)	1.75	0.665–4.601	0.257
	HDL-C (mmol/L)	0.083	0.012–0.587	0.013*
	LDL-C (mmol/L)	0.088	0.027–0.291	<0.001*

*Significant *P*-values (a *P*-value of less than 0.05).

## 4 Discussion

The TGF-β signaling pathway is currently a prominent area of research in the field of TAAD genetic pathogenesis. The first step in the activation of the TGF-β pathway activation is when the TGF-β ligand binds to TGFβR2, which initiates intracellular signal transduction through phosphorylation of SMADs, thereby resulting in the manifestation of a multitude of biological effects. Mutations in the TGF-β and its receptors alter the interactions and the consequent transduction in the TGF-β signaling pathway, with *TGF*β*R1/2* mutations [21,22,23] leading to impaired receptor activation and blockage of transmembrane signal transport, thereby affecting the expression and function of TGF-β1. In contrast, the majority of heterozygous and partial missense mutations have been identified in *TGFBR2* in patients with atypical MFS syndrome. Similarly, at least 8 *TGFBR1* mutations [19,20,22,23,24] and 27 *TGFBR2* mutations [16,17,18,19,20,22,23] have been detected in patients with LDS and FTAAD.

TGFBR2 was localized to the human chromosome 3p22. In a previous study, Scola examined a TAAD population and identified five SNPs belonging to the TGF-β pathway. The frequency of the AA GT of rs900 was observed to be low in TAAD patients, with the homozygous or heterozygous A allele appearing to exert a significant protective effect against the occurrence of TAAD [25]. As stated by Zuo et al. [32] and Staneviciute et al. [33], the TGFB1 rs1800469 TT GT was correlated to an increased risk of abdominal aortic aneurysm (AAA). As stated by Baas et al. [30] and Puchenkova et al. [31], the genetic variations in TGFBR1 rs1626340 and TGFBR2 rs1036095 have been linked to the development of AAA in the Dutch population. The TGFBR2 rs1036095 SNP is situated upstream of the TGFBR2 coding sequence. Our study identified that the GFs of TGFBR2 rs1036095 (*P* = 0.006) and AFs of TGFBR2 rs1036095 (*P* = 0.001) had a significant difference between the two groups. Additionally, the dominant model (CC + CG) GT of the TGFBR2 rs1036095 GT was found to be associated with an elevated risk of sTAAD. When we stratified participants according to sex, the GFs of TGFBR2 rs1036095 (*P* = 0.012) and AFs of TGFBR2 rs1036095 (*P* = 0.003) in women were detected to differ significantly among the two groups, indicating that, in all probability, a genetic variant of TGFBR2 rs1036095 is likely to increase the risk of TAAD in women.

FBN1 functions as a storage pool for TGF-β, with the quantity of TGF-β released into the body being contingent upon the content of this storage pool. In addition, mutations in FBN1 have been demonstrated to influence the level of TGF-β expression. A number of different mutations have been identified in the FBN1 gene. The occurrence of missense, frameshift, deletion/insertion, and early termination codon mutations leads to the generation of truncated FBN1 molecules, which are easily hydrolyzed by proteolytic enzymes, resulting in loss of microfibers, destruction of normal aortic vascular wall structure, and eventually the development of TAAD [26]. At present, there is evidence to suggest that more than 600 FBN1 mutations are likely related to the occurrence of MFS [8,26,27]. In a recent genome-wide analysis of sTAAD-related genes in European populations, Lemaire identified five FBN1 SNPs associated with sTAAD (rs2118181, rs1036477, rs10519177, rs755251, and rs4774517). The relationship between rs2118181 and TAAD was confirmed again in a case–control study conducted at Yale University, wherein C was identified as a risk allele of TAAD [28]. In the recent study of Chinese Han individuals, the FBN1 SNP rs2118181 polymorphism was found to be related to the sporadic aortic syndrome, with the C allele potentially being a protective factor against the development of the disease, which is contrary to the finding of previous studies on various populations [29]. Our study demonstrated that the GFs of FBN1 rs2118181 (*P* = 0.029) and AFs of FBN1 rs2118181 (*P* = 0.007) exhibited differential expression between the sTAAD and control groups. We also identified the FBN1 rs2118181 dominant model CC + CT GT (OR = 0.475; 95% CI: 0.272–0.830, *P* = 0.009) as an important risk factor for sTAAD development. Moreover, the C allele was considered to confer a protective factor against the occurrence of sTAAD, which is consistent with the aforementioned studies [29]. With regard to sexual dimorphism, we observed significant differences in the GFs of FBN1 rs2118181 (*P* = 0.001) and AFs of FBN1 rs2118181 (*P* = 0.001) in men between the sTAAD and control groups. Our findings confirmed that FBN1 rs2118181 is an increased risk factor for TAAD development in men.

Furthermore, significant differences were detected in the GFs of FBN1 rs1036477 (*P* = 0.029) and AFs of FBN1 rs1036477 (*P* = 0.007) between the two groups. Consequently, the FBN1 rs1036477 recessive model AA GT (OR = 2.105; 95% CI: 1.205–3.677, *P* = 0.009) was considered a risk factor for sTAAD. Concomitantly, notable discrepancies were observed in the GFs of FBN1 rs1036477 (*P* = 0.001) and AFs of FBN1 rs1036477 (*P* = 0.001) in male subjects between patients with sTAAD and healthy controls after stratification by sex. This suggested that men with the FBN1 rs1036477 polymorphism have an increased risk of developing TAAD. It is noteworthy that the GFs of TGFBR2 rs6785385 (*P* = 0.039) and FBN1 rs10519177 (*P* = 0.015), as well as the AFs of TGFBR2 rs6785385 (*P* = 0.012) and FBN1 rs10519177 (*P* = 0.005), exhibited significant differences between the two groups exclusively in men. This suggests that these two SNPs are independent risk factors for TAAD development in men.

Nonetheless, the present study is subject to certain limitations, given that it was conducted at a single center, and that its findings may not be applicable to other populations. To gain further insight into the correlation between GT and AFs of candidate genes and the risk of TAAD, it is necessary to carry out a multicenter study with a larger sample size in the future.

## 5 Conclusions

In conclusion, the current study identified that the *TGFBR2* rs1036095, *FBN1* rs1036477, and *FBN1* rs2118181 variations are associated with a genetic predisposition for the development of sTAAD in the Zhejiang Han population. Our study also recognized hypertension as well as TG, HDL-C, and LDL-C levels as risk factors for TAAD occurrence, consistent with the results of prior studies. After excluding confounding factors, TGFBR2 rs1036095 dominant model (CC + CG)/GG a danger factor for sTAAD development, suggesting that individuals carrying the TGFBR2 rs1036095 polymorphism are more likely to develop TAAD, especially for women. Moreover, our findings provided solid evidence that FBN1 rs1036477 recessive model AA GT and FBN1 rs2118181 dominant model CC + CT GT were correlated to an increased death rate in sTAAD men patients.
